# Negative inotropic mechanisms of β-cardiotoxin in cardiomyocytes by depression of myofilament ATPase activity without activation of the classical β-adrenergic pathway

**DOI:** 10.1038/s41598-021-00282-x

**Published:** 2021-10-27

**Authors:** Tuchakorn Lertwanakarn, Montamas Suntravat, Elda E. Sánchez, Beata M. Wolska, R. John Solaro, Pieter P. de Tombe, Kittipong Tachampa

**Affiliations:** 1 Department of Physiology, Faculty of Veterinary Science, Chulalongkorn University, Bangkok, Thailand; 2 National Natural Toxins Research Center, Texas-A&M University-Kingsville, Kingsville, TX USA; 3 Department of Chemistry, Texas A&M University-Kingsville, Kingsville, TX USA; 4 Department of Physiology and Biophysics, the University of Illinois at Chicago, Chicago, IL USA; 5 Department of Medicine, the University of Illinois at Chicago, Chicago, IL USA; 6 Phymedexp, Université de Montpellier, Inserm, CNRS, Montpellier, France

**Keywords:** Biochemistry, G protein-coupled receptors, Cardiology, Cardiovascular diseases, Physiology, Cardiovascular biology

## Abstract

Beta-cardiotoxin (β-CTX) from the king cobra venom (*Ophiophagus hannah*) was previously proposed as a novel β-adrenergic blocker. However, the involvement of β-adrenergic signaling by this compound has never been elucidated. The objectives of this study were to investigate the underlying mechanisms of β-CTX as a β-blocker and its association with the β-adrenergic pathway. The effects of β-CTX on isolated cardiac myocyte functions, calcium homeostasis, the phosphorylation level of targeted proteins, and the myofibrillar ATPase activity were studied. Healthy Sprague Dawley rats were used for cardiomyocytes isolation. Like propranolol, β-CTX attenuated the cardiomyocyte inotropy and calcium transient alterations as induced by isoproterenol stimulation. In contrast, these effects were not observed in forskolin-treated cells. Interestingly, cardiomyocytes treated with β-CTX showed no changes in phosphorylation level at any PKA-targeted sites in the myofilaments as demonstrated in Western blot analysis. The skinned fibers study revealed no change in myofilament kinetics by β-CTX. However, this protein exhibited the direct inhibition of myofibrillar ATPase activity with calcium de-sensitization of the enzyme. In summary, the negative inotropic mechanism of β-CTX was discovered. β-CTX exhibits an atypical β-blocker mechanism. These properties of β-CTX may benefit in developing a novel agent aid to treat hypertrophic cardiomyopathy.

## Introduction

Beta-cardiotoxin (β-CTX), is a non-enzymatic protein containing in the venom of the king cobra (*Ophiophagus hannah*)^1^. It contains 63 amino acids with a molecular weight of 7 kDa, approximately^1^. The protein is classified in the three-finger toxin (3FTx) family, constructed by five beta-strands folded up with four disulfide bonds^2^. Comparing the homology, β-CTX shows a structural similarity of 35–58% with other cobra cardiotoxins (CTXs)^2^. Unlike CTXs, β-CTX possesses no cytotoxic effect in myoblast and cardiomyoblast due to its unique structural properties^3^. Interestingly, when injected β-CTX intraperitoneally into rodents, a negative chronotropic effect is exhibited without changing the cardiac contractility index^1^. Since it expresses binding property to both β1 and β2 adrenergic receptors (β-ARs), β-CTX was previously proposed to be a potential novel β-blocking agent.

β-blocker (BB) is a class of drug widely used for many cardiovascular conditions including hypertension and hypertrophic cardiomyopathy (HCM). In hypertension, BBs decrease blood pressure via several mechanisms, including vasodilatation, decreased renin, and reduced cardiac output^4^. However, BBs are no longer recommended as first-line therapy for primary hypertension, due to their inferior outcome when compared to other antihypertensive agents^5^. Meanwhile, BB is recommended to use in HCM patients because of their mechanisms to inhibit the chronotropic and inotropic effect of the heart^4^. By reducing heart rate and attenuating contractility, left ventricular (LV) filling time will be prolonged ; hence, improving both the diastolic function and cardiac output. Up to date, only three BBs are reported to be mortality benefits in congestive heart failure (CHF) patients, including bisoprolol, carvedilol, and slow-released metoprolol^5–7^. However, no prospective data have shown the benefit of the current BBs on long-term outcomes in patients with non-obstructive HCM^6^. Therefore, due to these limitations, searching for new candidate BBs may benefit patients with hypertension and/or HCM.

In a classical-β-adrenergic pathway, β-agonist, such as isoproterenol (ISO), can bind to the β-AR and activate the cAMP-PKA-dependent downstream signalings in which promote phosphorylation of several targeted proteins including L-type calcium channel (LTCC), ryanodine receptor (RyR), and, phospholamban (PLN)^7^. As a positive inotropic mechanism of β-agonist, phosphorylation of these proteins by PKA increases calcium concentration in the sarcoplasm. Consequently, cross-bridges are activated and further enhance the contraction of the cardiomyocyte. On the other hand, inhibition of this pathway by BB such as propranolol (PP) would; therefore, result in a decreased phosphorylation status of these proteins, decreased the calcium transient, and attenuated the contraction^7^.

Interestingly, our recent study demonstrated the calcium-independent negative inotropic effects of β-CTX in the isolated cardiomyocyte^3^ (i.e., β-CTX reduced myocyte contractility without alteration in calcium transient at the basal state), indicating the possibility of involvement of a non-classical-β-adrenergic pathway by β-CTX. As a novel BBs candidate, the need of understanding molecular mechanisms is required to provide basic knowledge for the drug developmental process. Therefore, to elucidate the underlying mechanism(s) of β-CTX on depressing cardiomyocyte function, the involvement of β-adrenergic signaling (β-AS) and the direct effect of the compound on isolated myofibrils were investigated. The objectives of the study were (i) to determine the effects of β-CTX on isolated cardiomyocyte functions with the presence of isoproterenol (ISO), the standard β-agonist, and forskolin (FSK), the adenylyl cyclase activator, (ii) to evaluate the alteration of phosphorylated proteins responsive in the β-adrenergic pathway and (iii) to study the direct effects of the compound on myofilament Ca^2+^-sensitivity and enzyme activity.

## Results

### β-CTX suppressed cardiac functions and peak Ca^2+^ transient in ISO-induced cardiomyocytes

**Figure 1:**
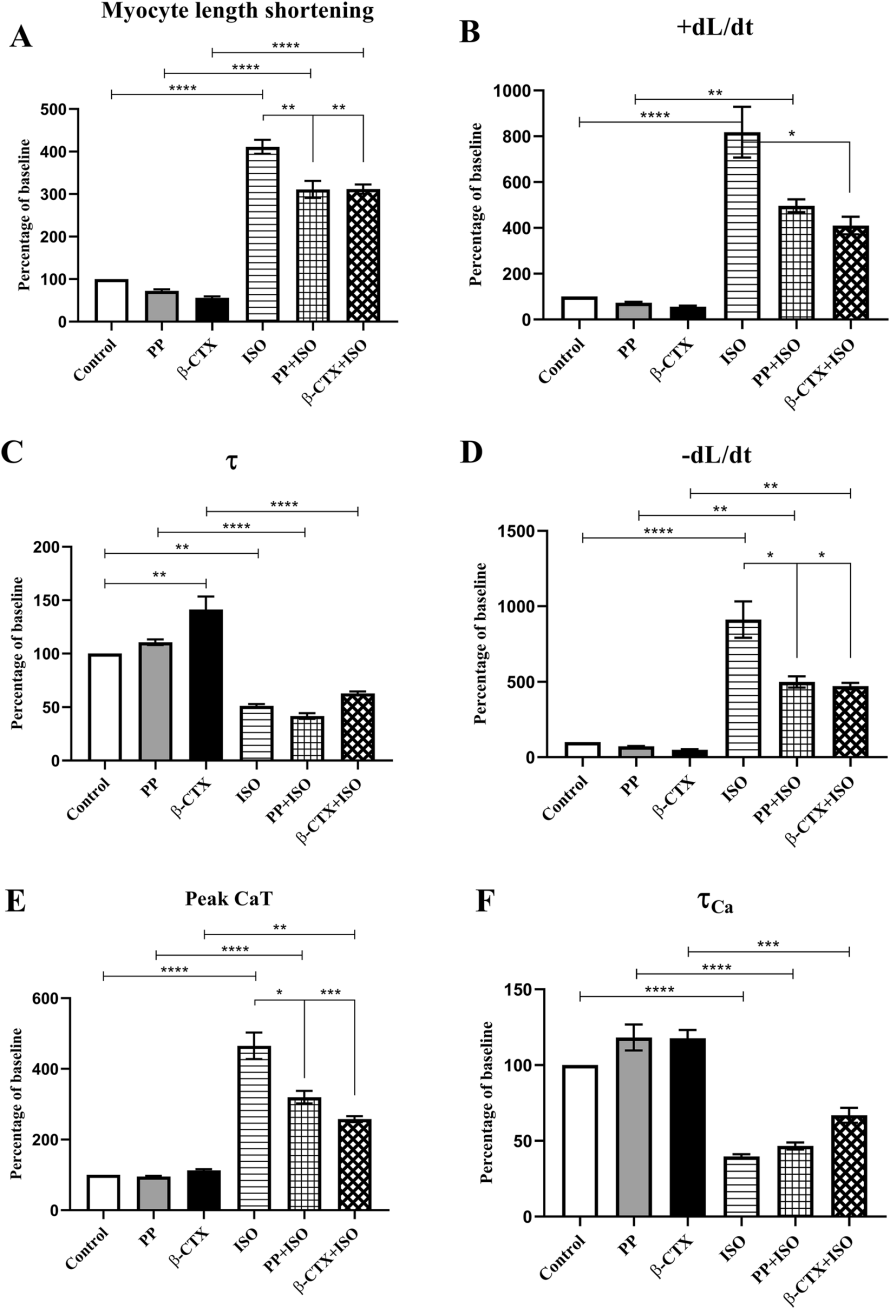
Bar graphs representing cardiac parameters comparing between cells perfused with control solution, propranolol only (PP), β-CTX only, isoproterenol only (ISO), PP + ISO, and β-CTX + ISO (n = 6 each). The cardiomyocyte properties are shown as percentage changes from baseline of (**A**) myocyte length shortening, (**B**) shortening velocity; + dL/dt, (**C**) relaxation index; τ, (**D**) re-lengthening velocity; -dL/dt, (**E**) peak calcium transient (CaT), and calcium decaying time (τ_Ca_). Data are represented in mean ± S.E.M. * *p* < 0.05, ** *p* < 0.01, *** *p* < 0.001, **** *p* < 0.0001.

The effects of β-CTX on ISO-induced cardiomyocytes are represented in Fig. 1 and supplementary figure S1. Averaged data for Fig. 1 are provided in Table S1 in the supplementary material. Figure 1A,B demonstrates inotropic parameters, the percentage change of baseline of cell length shortening, and the shortening velocity (+ dL/dt), respectively. At the basal state, there was no significant difference in both inotropic parameters among cells treated with control, propranolol (PP), and β-CTX. In the ISO-stimulating condition, the β-agonist drastically promoted the myocyte length shortening (Fig. 1A) when comparing between ISO and control (417% and 100%, *p* < 0.0001), PP + ISO and PP (310% and 72%; *p* < 0.0001), and β-CTX + ISO and β-CTX (319% and 56%; *p* < 0.0001). Similarly, ISO also enhanced the + dL/dt of the myocyte (Fig. 1B) when comparing between ISO and control (817% and 100%; *p* < 0.0001), as well as PP + ISO and PP (497% and 73%; *p* < 0.01). Pre-incubation of PP before ISO perfusion significantly attenuated the stimulatory effects of ISO on myocyte length shortening (76% *p* < 0.01) (Fig. 1A). The effect was also presented in β-CTX + ISO group which reduced the cell length shortening (76%; *p* < 0.01) and + dL/dt (50%; *p* < 0.05%) comparing to ISO-treated cells (Fig. 1A,B).

Cardiac relaxation parameters are demonstrated in Fig. 1C,D as relaxation index (τ) and re-lengthening velocity (-dL/dt), respectively. At the basal condition, β-CTX significantly prolonged the τ index comparing to control (141% and 100%; *p* < 0.01; Fig. 1C) without affecting -dL/dt (Fig. 1D). Figure 1C demonstrates the stimulatory effect of ISO as indicated by the reduction of the τ index when comparing between ISO and control (51% and 100%; *p* < 0.01), PP + ISO and PP (42% and 111%; *p* < 0.0001), and β-CTX + ISO and β-CTX (63% and 141%; *p* < 0.0001). Likewise, ISO also enhances the velocity of relaxation (Fig. 1D) when comparing between ISO and control (906% and 100%; *p* < 0.01), PP + ISO and PP (499% and 72%; *p* < 0.01), and β-CTX + ISO and β-CTX (473% and 49%; *p* < 0.01). Interestingly, pre-incubation with neither PP nor β-CTX could change the relaxation index in the presence of ISO (Fig. 1C); whereas PP and β-CTX potentially attenuated the -dL/dt in ISO-induced condition for 55% and 52%, respectively (*p* < 0.05) (Fig. 1D).

Intracellular Ca^2+^ profiles are displayed in Fig. 1E,F as peak calcium transient (CaT) and calcium decaying index (τ_Ca_), respectively. Figure 1E demonstrates no difference among control, PP, and β-CTX-treated cells at the basal condition. Activating the cells with ISO represents markedly elevation in the Ca^2+^ transient when comparing between ISO and control (467% and 100%; *p* < 0.0001), PP + ISO and PP (320% and 95%; *p* < 0.0001), and β-CTX + ISO and β-CTX (258% and 113%; *p* < 0.01). Cells pre-incubated with PP significantly attenuated the ISO effects on peak CaT (69%; *p* < 0.05). Likewise, the effect was also presented in the β-CTX + ISO group which alleviated the maximized CaT (*p* < 0.001). Focusing on τ_Ca_, there was also no difference between control, PP, and β-CTX-treated cells. However, ISO-treated cells significantly shortened the τ_Ca_ when comparing to control (40% and 100%; *p* < 0.0001), PP + ISO and PP (47% and 118%; *p* < 0.0001), and β-CTX + ISO and β-CTX (67% and 118%; *p* < 0.001). No effects were observed in PP + ISO and β-CTX + ISO when comparing to the ISO-treated cells. Results indicating that β-CTX could reduce cardiac functions, as well as the Ca^2+^ homeostasis in ISO-induced cardiomyocytes.

### β-CTX had no impact on FSK-induced cardiomyocytes

**Figure 2:**
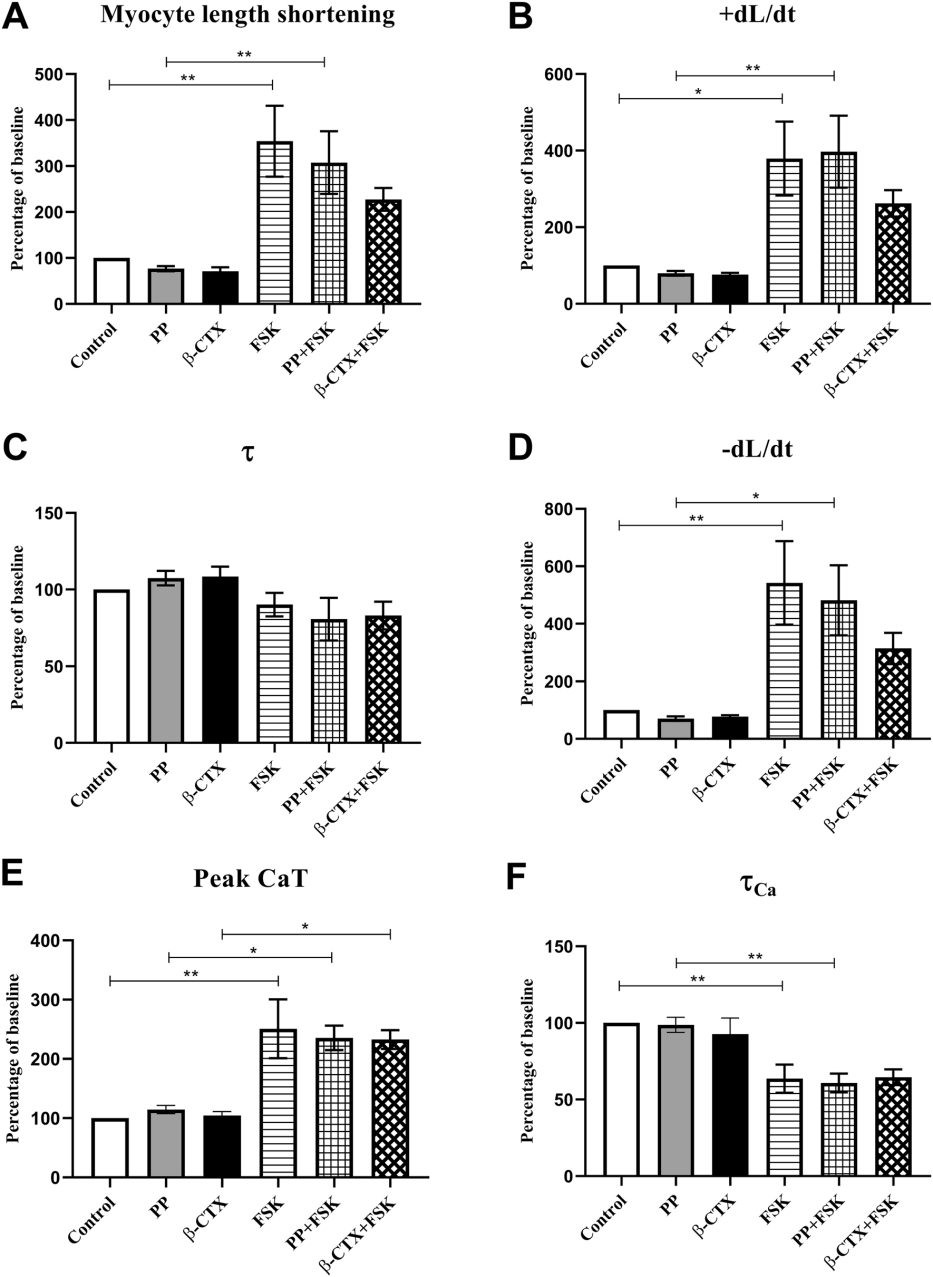
Bar graphs representing cardiac parameters comparing between cells perfused with control solution, propranolol only (PP), β-CTX only, Forskolin only (FSK), PP + FSK, and β-CTX + FSK (n = 6 each). The cardiomyocyte properties are shown as percentage changes from baseline of (**A**) myocyte length shortening, (**B**) shortening velocity; + dL/dt, (**C**) relaxation index; τ, (**D**) re-lengthening velocity; -dL/dt, (**E**) peak calcium transient (CaT), and calcium decaying time (τ_Ca_). Data are represented in mean ± S.E.M. * *p* < 0.05, ** *p* < 0.01.

To investigate the involvement of the β-AS signaling pathway, FSK was used to stimulate adenylyl cyclase and to increase the level of cAMP. Figure 2 and supplemental Figure S1 represent the effects of β-CTX on FSK-induced cardiomyocytes. Averaged data of Fig. 2 are provided in the supplemental Table S2. The inotropic effects are demonstrated in Fig. 2A,B as myocyte length shortening and + dL/dt, respectively. Similarly, there were no changes induced by both PP and β-CTX at the basal condition. Figure 2A shows the drastic elevation of myocyte length shortening in cells treated with FSK comparing to control (353.9% and 100%; *p* < 0.01), and PP + FSK comparing to PP (307% and 77%; *p* < 0.01). The effect was also represented in + dL/dt (Fig. 2B), where FSK promoted the rate of length changes when comparing between FSK and control (379% and 100%; *p* < 0.05), and PP + FSK and PP (397% and 80%; *p* < 0.01). There were no alterations when comparing β-CTX + FSK with FSK and PP + FSK group in both parameters. Off-note, it seems that effects of myocyte shortening, + dL/dt, and -dL/dt of cardiomyocytes pre-treated with β-CTX tend to decrease from the FSK-induction; however, there were not statistically different (*p*-value > 0.05).

The relaxation index and the relaxation velocity are also shown in Fig. 2C,D respectively. Interestingly, there was no difference of τ among groups regardless of the FSK-stimulating condition (Fig. 2C). Whereas the stimulatory effect of FSK could still be observed in -dL/dt (Fig. 2D). While there was no difference among cells treated with control, PP, and β-CTX, perfusion with FSK could enhance the rate of relaxation comparing between FSK and control (542% and 100%; *p* < 0.01), and PP + FSK and PP (481% and 70%; *p* < 0.05). Likewise, β-CTX did not show any effect on these lusitropic parameters.

Ca^2+^-homeostasis profiles are displayed in Fig. 2E and 2F as peak CaT and τ_Ca_, respectively. There was no alteration in peak CaT among control, PP, and β-CTX at the basal condition (Fig. 2E). Introducing FSK to the cardiomyocytes significantly elevated the CaT comparing between FSK and control (251% and 100%; *p* < 0.01), PP + FSK and PP (236% and 115%; *p* < 0.05), and β-CTX + FSK and β-CTX (233% and 104%; *p* < 0.05). On the other hand, the τ_Ca_ index represented in Fig. 2F exhibited that the FSK also shortened the parameter when comparing between FSK and control (64% and 100%; *p* < 0.01), and PP + FSK and PP (61% and 99%; *p* < 0.01). However, β-CTX, on the contrary, had no impact on cardiac functions and calcium homeostasis in FSK-induced myocytes. Taken together, the action of β-CTX was similar to propranolol action where the inhibitory effects on cardiomyocytes were overridden by the stimulation with forskolin.

### β-CTX did not change the phosphorylation of proteins involved in β-adrenergic signaling

**Figure 3:**
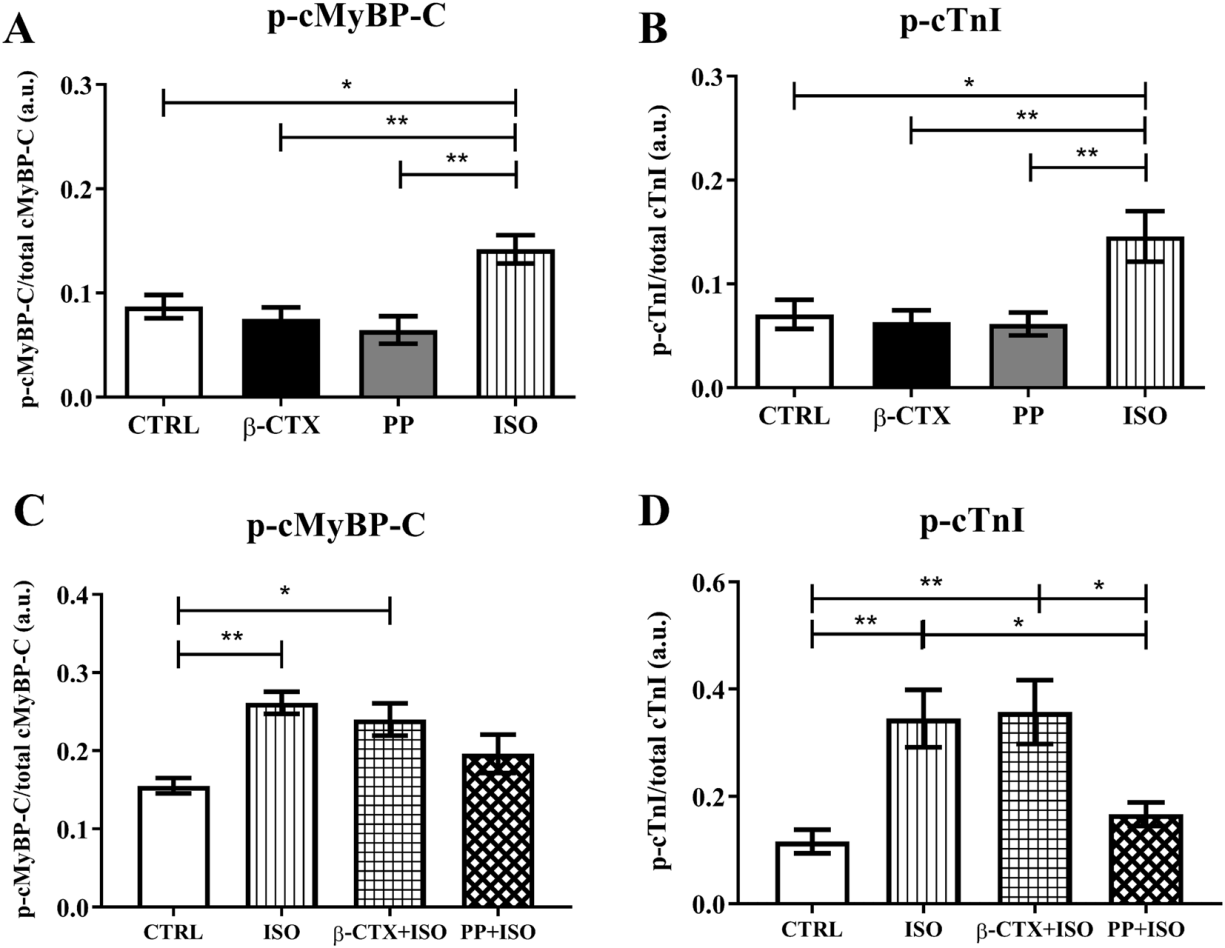
Results from phosphor-staining of cardiac myofilament comparing phosphorylation level of (**A**) cMyBP-C and (**B**) cTnI at the basal condition, and (**C**) cMyBP-C and (**D**) cTnI, in ISO-induced condition (n = 6 each). The full-length gels are provided in the supplemental file (Figure S2). Data are shown in mean ± S.E.M. Control (CTRL), Propranolol (PP), Isoproterenol (ISO), *p < 0.05, ** *p* < 0.01.

It is well known that the downstream signaling of β-AR is associated with the activation of PKA. If β-CTX is a classical β-blocker, we expected to see an alteration of protein phosphorylation in ISO stimulating conditions. To prove this speculation, we, therefore, further investigated the involvement of the β-AS signaling pathway. Common targets of PKA phosphorylation proteins were selected (i.e., cardiac myosin binding protein-C and cardiac troponin I) then electrophoresis of these proteins was performed. The ProQ® phosphor-staining was applied on ventricular myocytes receiving different treatments are shown in Fig. 3 and supplementary figure S2. Descriptive information of data in Fig. 3 was reported in table S3. Obviously, only cardiac myosin binding protein-C (cMyBP-C) and cardiac troponin I (cTnI) were the only two among all myofilament proteins which significantly increased their phosphorylation after ISO incubation (Fig. 3A,B; *p* < 0.05). Comparing to the ISO, pre-treated cells with β-CTX could not attenuate the stimulatory effect, whereas the PP + ISO group, promisingly, showed a significant reduction of phosphorylation on both cMyBP-C and cTnI (Fig. 3C,D). There was no difference in phosphorylation of other myofibrillar proteins (desmins, cTnT, Tm, MLC2) among groups (Figure S2 and Table S3).

**Figure 4:**
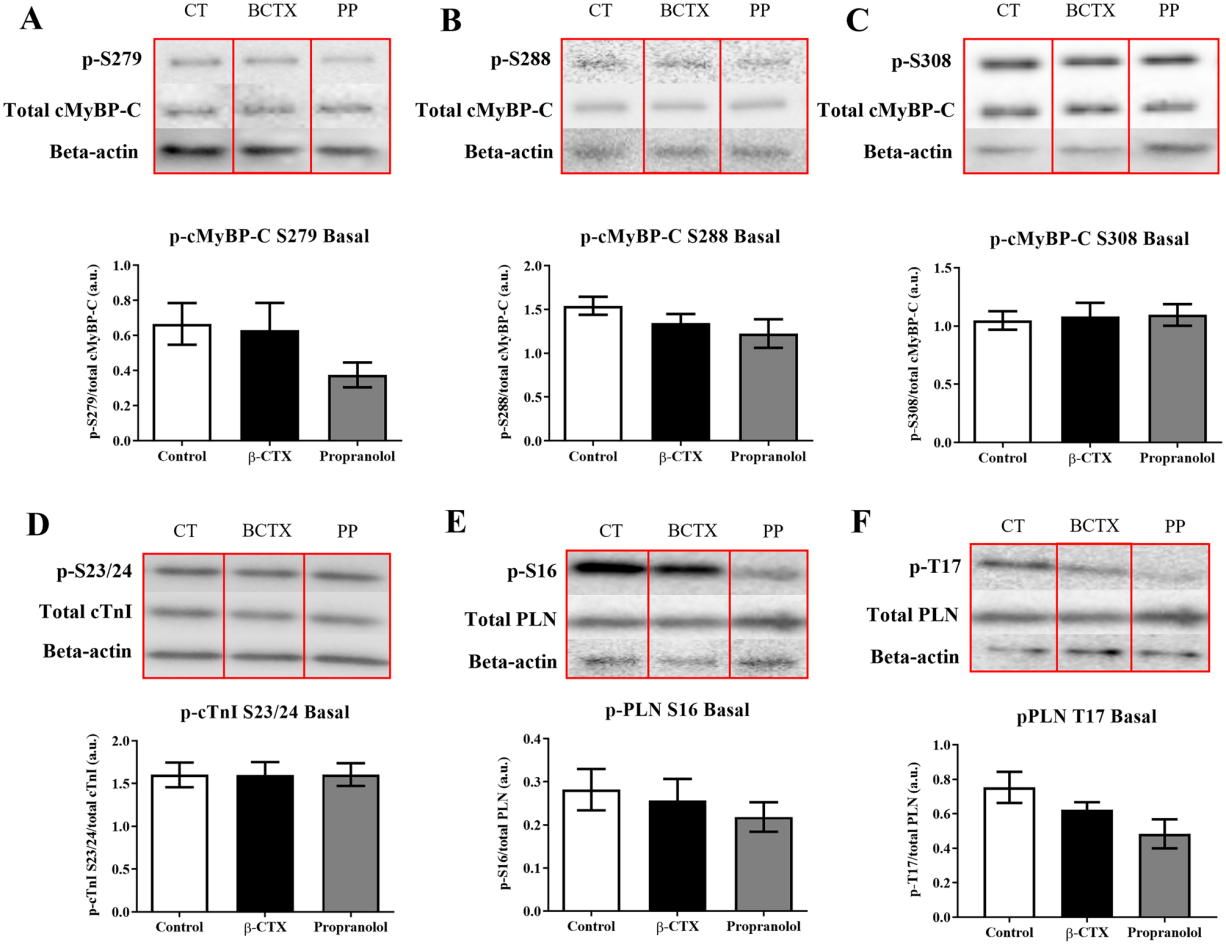
Comparison of specific phosphorylation sites of proteins involving in β-adrenergic signaling after incubated cardiomyocytes with control solution, β-CTX, and propranolol only (PP) (n = 6 each). The grouping of gels/blots cropped from different parts of the same gel. Uncropped blots are available in supplementary (Figure S5 and S6). Representative cropped blots are shown above their corresponding bar graphs. Data are demonstrated in mean ± S.E.M. of the (**A**) p-S279, (**B**) p-S288 and, (**C**) p-S308 of cardiac myosin binding protein-C (cMyBP-C), (**D**) p-S23/24 of cardiac troponin I (cTnI), and (G) p-S16 and (H) p-T17 of phospholamban (PLN).

**Figure 5:**
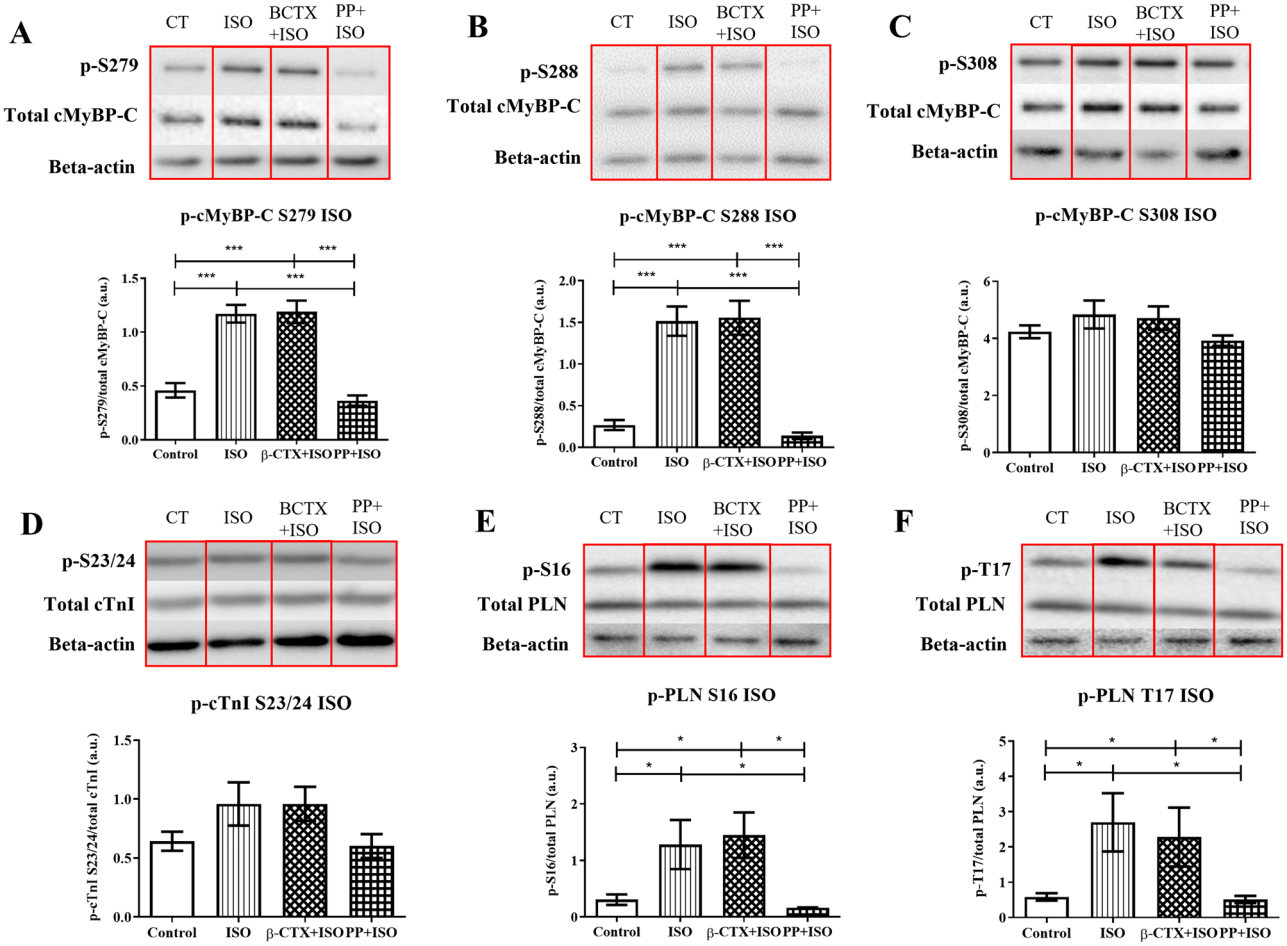
Comparison of specific phosphorylation sites of proteins involving in β-adrenergic signaling after incubated cardiomyocytes with control solution, ISO only, PP + ISO, and β-CTX + ISO (n = 6 each). The grouping of gels/blots cropped from different parts of the same gel. Uncropped blots are available in supplementary (Figure S6 and S7). Representative cropped blots are shown above their corresponding bar graphs. Data are demonstrated as mean ± S.E.M. of the (**A**) p-S279, (**B**) p-S288 and (**C**) p-S308 of cardiac myosin binding protein-C (cMyBP-C), (D) p-S23/24 of cardiac troponin I (cTnI), and (G) p-S16 and (H) p-T17 of phospholamban (PLN). * *p* < 0.05, *** *p* < 0.001.

In addition, alteration in specific phosphorylated sites was evaluated at basal state (Fig. 4) and ISO-induced cardiomyocyte (Fig. 5). The descriptive data was shown in supplemental tables S4 and S5. Without ISO-stimulation, there were no changes of any PKA-phosphorylated sites, including Ser279, Ser288, and Ser308 of cMyBP-C, Ser23/24 of cTnI, and Ser16 and Thr17 of phospholamban (PLN) among control, PP-, and β-CTX-treated cells (Fig. 4. A-F). In the ISO-stimulation condition, the phosphorylations of cMyBP-C at Ser279, Ser288, and Ser308 were presented in Fig. 5A–C. In ISO-treated cells, the level of phosphorylation increased significantly at Ser279 and Ser288 whereas the level of Ser308 was similar to that in the control group. The cells receiving β-CTX + ISO showed similar phosphorylation levels as the same as the ISO group, whereas pre-incubating myocytes with PP blocked the effect of ISO as shown by the lower expression on the two phosphorylation sites (Fig. 5A,B; *p* < 0.05). The phosphorylation site of cTnI at Ser23/24 was also investigated in ISO-inducing conditions (Fig. 5D). Results demonstrated no variations between treatments on these phosphorylation sites. The phosphorylation of PLN at both Ser16 and Thr17 sites were also determined in Fig. 5E,F, respectively. A similar pattern was shown on both phosphorylation sites. Ventricular cells incubating with either ISO alone or β-CTX + ISO showed an elevation of both Ser16 and Thr17 sites comparing to control (*p* < 0.05). Contrarily to β-CTX, the PP + ISO group significantly reduced the p-Ser16 and Thr17 indicates the blockade of the β-AR (*p* < 0.05). These results demonstrated that β-CTX was not associated with the phosphorylation of proteins targeted in β-AR stimulation.

### β-CTX did not change the Ca^2+^-sensitivity of isometric force contraction whereas shifting the Ca^2+^-responsiveness of actomyosin ATPase activity

**Figure 6:**
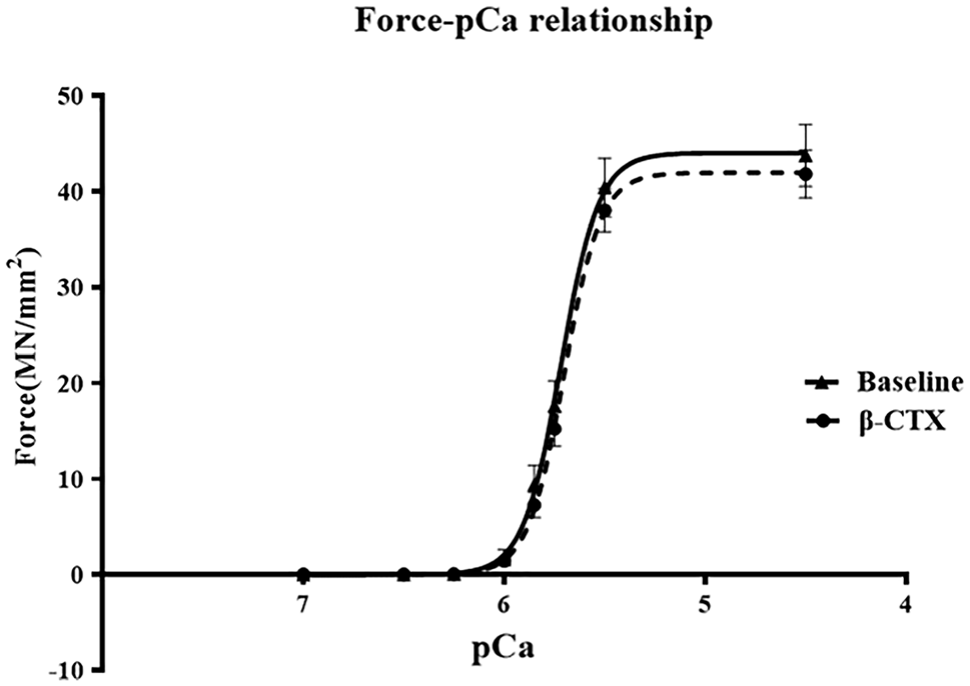
The sigmoidal curve represents the force-pCa relationship in the detergent extracted “skinned” fiber experiment (n = 11 fibers) comparing at baseline and after incubated with β-CTX. Data are shown as mean ± S.E.M.

Since β-CTX did not change the alteration of the PKA phosphorylation sites, the mechanism of this compound may act via a different pathway. Thus, the direct effect of β-CTX on myofilament dynamic and kinetic was further investigated. The alteration in Ca^2+^-sensitivity of the cardiac skinned myofibers was evaluated so as to explain the negative inotropic effects by β-CTX. Table 1. presents the data compared to a baseline of the maximal tension generated, pCa_50,_ and Hill’s coefficient (n_*H*_). The force-pCa relationship is shown in Fig. 6. The tension of the myofibers created after exposure to β-CTX (42 MN/mm^2^) was not significantly changed from the baseline (44 MN/mm^2^) (*p* > 0.05). Similarly, the pCa_50_ and the n_*H*_ from the experiment were not significantly altered compared to the baseline measurement (Table 1). This result indicated that there was no direct effect of β-CTX on cardiac myofilament kinetics.

**Table 1:** Comparisons of maximal tension, pCa_50,_ and Hill’s coefficient (n_*H*_) in detergent-extracted (skinned) fibers before and after receiving β-CTX (n = 9).

	Before	After	*p*-value
Maximal tension	44.04	41.96	> 0.05
pCa_50_	5.717	5.702	> 0.05
n_*H*_	−4.600	−4.779	> 0.05

**Figure 7:**
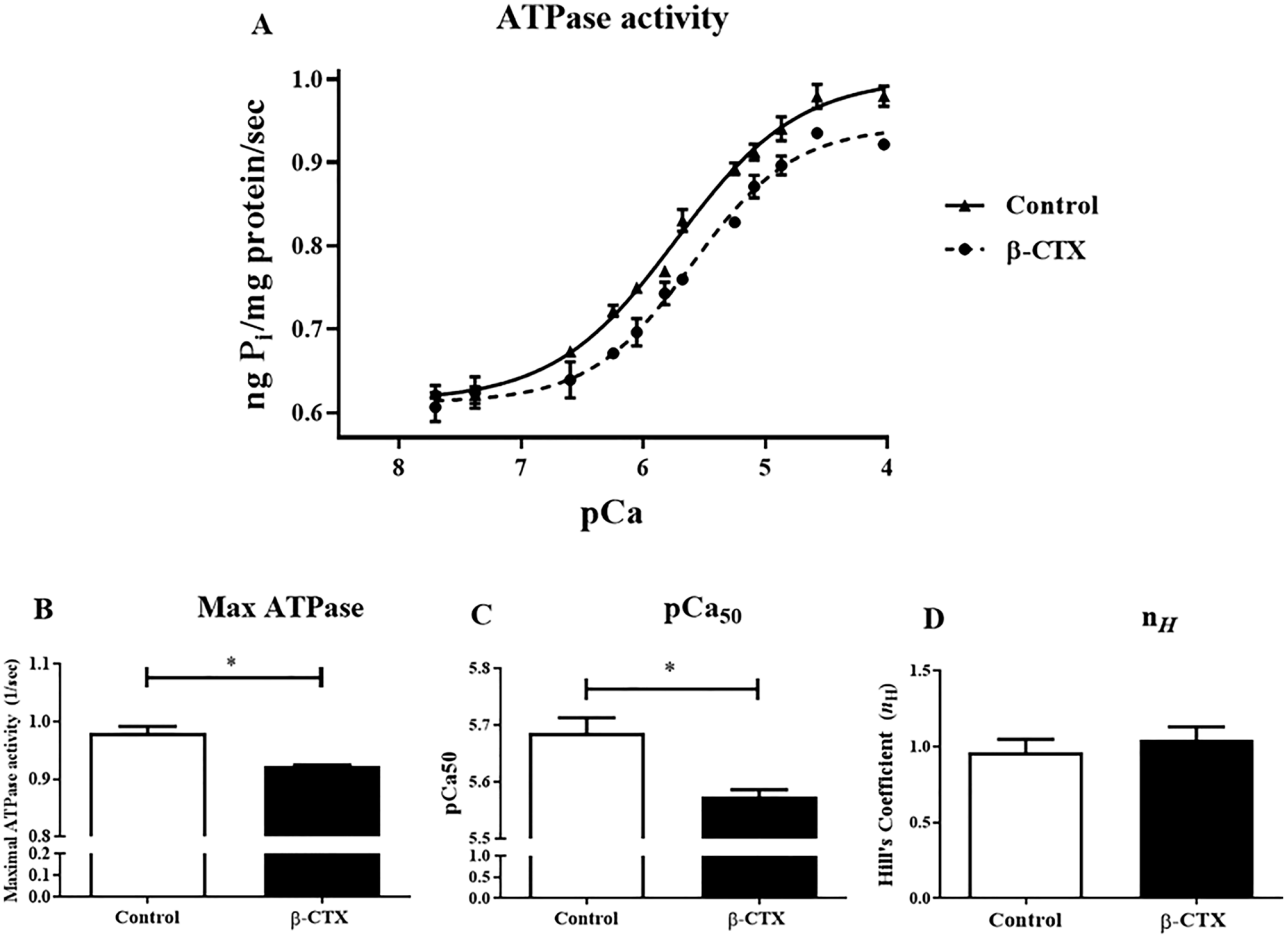
Acto-myosin ATPase activity was monitored in isolated myofibril using the malachite green assay. (**A**) The sigmoidal curve represents the pCa-ATPase activity relationship comparing between control (n = 6) and β-CTX-treated myofibrils (n = 5); whereas bar graphs show the comparison of the (**B **) maximal activity, (**C**) pCa_50_, and (**D**) and Hill’s coefficient (n_*H*_), respectively, between control and β-CTX groups. * *p* < 0.05.

We further investigated the direct of β-CTX on the myofilament enzyme activity by measured the actomyosin ATPase activity using a malachite green assay and plotted the relationship of pCa-ATPase activity as shown in Fig. 7. The reduction in Ca^2+^-responsiveness of the enzyme activity was noticed as expressed by the right-shifted of the Ca^2+^-ATPase activity plot (Fig. 7A). The sigmoidal curve fit revealed that the half-maximal activity concentrations (pCa_50_) of myofibril receiving 1 µM of β-CTX (5.57 ± 0.01) were lesser than control (5.69 ± 0.03), significantly (Fig. 7C). Moreover, Fig. 7B demonstrated the reduction of the maximal ATPase activity (at pCa = 4.03) of myofibrils receiving β-CTX comparing to the control group (0.92 ± 0.003 vs. 0.98 ± 0.012 mol P_i_/s; *p* < 0.05). However, Hill’s coefficient (n_*H*_) from the study showed unremarkable changes (Fig. 7D).

## Discussion

Little is known about the mechanisms of action of β-CTX. Up to date, there are only three publications regarding the β-CTX in the PubMed database. β-CTX was shown to bind to both β_1_- and β_2_-adrenergic receptors in vitro with the negative inotropic effect in vivo^19^*.* However, an ex vivo study from the same research group revealed an unchanged in the systolic function parameter. Therefore, β-CTX was proposed as a novel β-blocking agent. We have published recently and demonstrated that the effect of β-CTX exclusively occurs in striated muscle cell lines^11^. In addition, β-CTX could reduce inotropic parameters (i.e., cell length shortening and + dL/dT) without changing the amplitude of calcium transient^11^. However, prolongation of calcium decay was observed^11^. We hypothesized that β-CTX may not act via the classical β-adrenergic signaling pathway. To prove our hypothesis, the current study was designed to examine stepwise the effect of β-CTX on cardiomyocyte functions under β-AR stimulation by ISO and/or FSK. We found that β-CTX could attenuate the positive inotropic effect of ISO with a magnitude higher than a classical β-blocking agent, propranolol. In addition, the biochemical study revealed that β-CTX does not change the protein phosphorylation state of any major targeted proteins of β-AR stimulation. These results clearly point out that the negative inotropic effect of β-CTX was not through a classical β-AS. Finally, we further demonstrated the molecular mechanism of β-CTX in which directly suppresses actomyosin ATPase activity and decreases the calcium sensitivity of the myofilaments.

In our first experiment, ISO or FSK was introduced to the cardiomyocytes to mimic the stimulation of the β-AS or the activation of the AC. As expected, our findings demonstrated that both the β-agonist and the AC activator promoted the inotropic and lusitropic responses in isolated cardiomyocytes. During both conditions, the positive inotropy was associated with the elevation of the amplitude of the CaT; whereas, the τ_Ca_ was not altered regardless of the negative lusitropy. It is well known that the β-ARs are coupled with the G-proteins. After the receptor is bound to the β-agonist, G-protein subunit α_s_ would further stimulate the AC function, turning ATP into cAMP, and switch on the cAMP-PKA pathway^8^. As an active enzyme, PKA consequently phosphorylates other Ca^2+^-handling proteins (such as LTCC, RyR, and PLN), to promote the elevation of intracellular Ca^2+^^9^, and myofilament proteins (notably, cMyBP-C and cTnI), to accelerate myofibrillar dynamics^10,11^. Interestingly, our findings revealed that β-CTX attenuated the stimulatory effect of ISO in most parameters. The deterioration effects of the myocyte functions, inotropy, and lusitropy by β-CTX were consistent with those found in the basal condition as in previous study^3^. However, the blunting effect of the peak CaT was found only in the ISO-stimulating condition. We postulated that the ISO-induction would amplify the sensitivity in detecting β-blocking effects of β-CTX as previously described^12^. Moreover, the decrease of Ca^2+^-decaying (τ_Ca_) reflexes the delay in Ca^2+^ uptake by SERCa. Since phosphorylation of PLN was unaltered, thus, we speculated that the β-CTX might attenuate directly the SERCa activity. Nevertheless, the effect of β-CTX on intracellular Ca^2+^ mobility during ISO stimulation needs further investigation. Other possible explanations for this observation including the direct inhibition of LTCC, COX-2-dependent pathway, other GPCRs in which cooperated to phospholipase C, and direct activation of eNOS^13–16^. These mechanisms may decline intracellular Ca^2+^ and thereby suppressing myofilament activity. In addition, we found a different inhibitory level between propranolol and β-CTX on some parameters including + dL/dt and peak CaT. This result suggests that this toxin may non-competitively bind to the β-ARs. However, the exact binding residues of the compound on the β-ARs are not fully studied yet. It is believed that the 3FTX conformation associated with some specific residues is responsible for this binding, as evidenced by a previous study in which the synthesized single strand peptide of β-CTX does not interact with either β-1 or -2 ARs^1^.

It is generally known that β-AR predominantly cooperates with the AC activity, and cAMP-PKA pathway^17^. However, one of our key findings demonstrated that pre-incubation of β-CTX in the FSK-induced cells had no effects on inhibiting the AC activity**.** These results indicated that β-CTX’s action may not mediate via the classical β-AS. Although PP is a known β-blocking agent which reduces the AC activity^18^; however, the effects that occurred in this study were not clearly observed. We speculated that the concentration of PP used in the study was not reached its pharmacological action. Still, these findings supported the idea that the β-blocking property of β-CTX does not mediate the AC activity.

To further strengthen our idea that the β-CTX action is not mediated through the β-AS pathway, we employed the protein modification by phosphorylation study. Within the authors' knowledge, this is the first study to test whether the snake toxin mediates through the β-adrenergic pathways. As expected, ISO-treated cells augmented phosphorylation levels in cMyBP-C and cTnI comparing to control on the myofilament phosphor-staining gel. The β-agonist modestly activates the cAMP-PKA by promoting the phosphorylation of cMyBP-C at Ser279 and Ser288, cTnI, and PLN at Ser16 and Thr17^19–21^. In contrast, the pre-incubation of PP exhibited the β-blockade activity by attenuating all responsive phosphorylation sites activated by ISO. Consistently, the reduction in PKA-phosphorylated sites is also found in other BBs, such as landiolol, metoprolol, and carvedilol, where the p-Ser2808 of ryanodine receptor (RyR), as well as Ser-16 and Thr-17 of p-PLN, are reduced^19,20,22^. On the other hand, β-CTX did not show any impact on PKA-phosphorylation sites of cMyBP-C, cTnI, and PLN. Again, these findings strengthened our hypothesis that the mechanism of the β-CTX was not involved with the cAMP-PKA signaling, literally through non-classical the β-AS. Although there are some limitations in this experiment in which we did not test the phosphorylation level of all other targeted proteins for PKA such as the LTCC, RyR2, and the small-conductance Ca^2+^-activated channel K^+^ channel (SK)^23–26^; however, the major PKA targets were selected and our findings may sufficient to conclude that β-CTX was unlikely exerted classical β-blocking activity as previously described by Rajagopalan and colleagues^1^.

Since β-CTX is shown to inhibit myofibrillar ATPase, the question has been raised whether this protein is cell-permeant? The answer to this question is to await further investigation. However, pieces of indirect evidence would presumably indicate that β-CTX may also possess membrane translocation properties. Firstly, β-CTX belongs to the 3FTxs family in which shared the overall structural similarity with other cardiotoxins (CTXs) in the conserved domains^27^. Accumulating evidence has suggested that the biological activities of the cardiotoxin (CTX) are not totally dependent only on direct plasma membrane interaction but also involve modulation of intracellular signaling and cell penetration^28–31^. Previous reports demonstrated that CTXs isolated from *N. atra*, *N. kaouthia*, *N. oxiana*, and *N. mossambica* with rhodamine-labeled, showed the membrane translocation and interacted with cytosolic lysosome and mitochondria^28–31^. Secondly, an AI-sequence-based predictor tool for identifying cell-penetrating peptides revealed that residues 22–33 of β-CTX (i.e., CVKMTIKKLPS) have a high confidential score (> 70%) indicating a high probability of cell-penetrating by this protein (http://server.malab.cn/CPPred-FL/ProcessServlet2). Data shown in our study have also strengthened this idea in which we demonstrated that β-CTX also directly inhibited the actomyosin ATPase activity without altering the β-AR pathways. Up to date, none of the other snake venom toxins exhibit the ATPase inhibitory property. The myofibril enzyme activity is well-known for its role in the cross-bridge cycling rate as well as the velocity of the fibers^32,33^. Thus, we speculated that the blunting of the ventricular myocyte contractility by β-CTX may mediate through this mechanism. However, it was interesting that although the Ca^2+^-sensitivity of the myofiber enzyme activity was decreased, still, the Ca^2+^-sensitivity to generate the isometric force by the fibers remained constant. As previously described, the alteration in ATPase activity is not correlated with the P_0_ tension in the isometric condition^34^. The other BBs (i.e., carvedilol and metoprolol) in which act through the cAMP-PKA pathway, showed no effect on actomyosin ATPase activity and Ca^2+-^sensitivity^35–37^.

The reduction in myofilament ATPase activity was also found by other compounds such as blebbistatin, N-benzyl-*p*-toluene sulphonamide (BTS), 2,3-butanedione monoxime (BDM), and Mavacamten^38–41^. Interestingly, the latter is a novel compound that has shown a beneficial effect in HCM patients as shown by recent clinical trials^42,43^. Since β-CTX also exerts ATPase inhibitory effect, therefore; it would be interesting to develop this protein as a new drug aid to treat HCM patients. However, further investigation needs to augment more understanding about this protein such as pharmacokinetic and pharmacodynamic properties of this protein.

It is widely known that not only the phosphorylation of the myosin regulatory light chain regulates the actomyosin cross-bridge^44,45^, the myofilament Ca^2+^-sensitivity, and force generation but also contributed by the phosphorylation of cMyBP-C and cTnI^46,47^. However, our study suggested that β-CTX did not alter any of myofilament protein phosphorylation. Therefore, we speculate that β-CTX may also allosterically blocking the myosin ATPase site and disrupt the equilibrium state of the cross-bridge. A reduction in ATPase activity may then result in the inhibition of ventricular myocyte shortening and the myofibrillar dynamics.

Regulations of myocardial function and calcium homeostasis are complex processes and involve different types of proteins including ion channels. The effect of β-CTX on cardiac ion channels has never been investigated. β-CTX has also posses a negative chronotropic effect in the in vivo experiment^9^. We; hence, speculated that β-CTX may exert pharmacological actions on some cardiac ion channels such as Hyperpolarization-activated cyclic nucleotide-gated (HCN) channels, LTCC, SERCa, or other receptor-mediated pathways. Further electrophysiological studies are required in order to fully understand this toxin.

**Figure 8:**
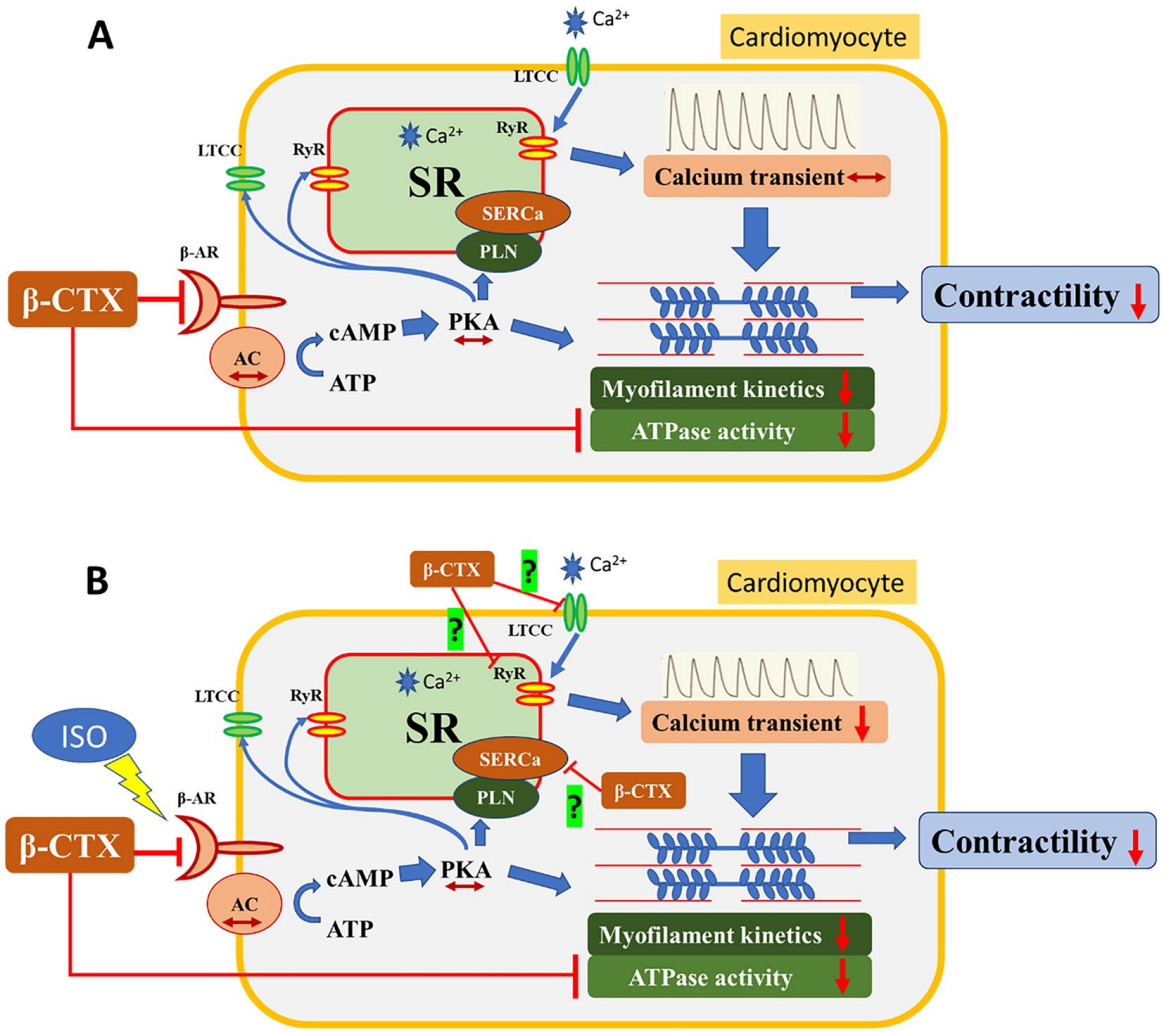
The diagram illustrates the proposed mechanisms of action of β-CTX. The β-CTX may non-competitively block the β-adrenergic receptor (β-AR) without affecting the downstream signaling. (A) Under the non-stimulating condition, the β-CTX directly reduces the myofibrillar ATPase activity, resulting in the reduction of myofilament kinetics and contractility. The calcium transient is, therefore, unaffected. (B) Under the stimulating condition with ISO, the cAMP-PKA pathway, however, could not be activated. The calcium transient is decreased likely due to the inhibition of SERCa. The effect of β-CTX on LTCC and RyR is unknown and awaits further investigation.

In conclusion, the novel molecular mechanism of action of β-CTX has now been proposed in Fig. 8. The action of β-CTX did not mediate through the classical β-AS and cAMP-PKA pathway as originally proposed. In addition, the diminishing of cardiac functions induced by the protein was due to the inhibition of myofilament ATPase activity. Understanding the precise mechanism of this protein may lead to the development of a new cardiovascular agent aids to treat HCM in the future.

## Materials and methods

### Ethical approval

This study was carried out in strict accordance with the Guide for the Care and Use of Laboratory Animals (NIH Publication, 8th Edition, 2011). All animal experiments and protocols were approved by the University of Illinois at Chicago Animal Care and Use Committee (ACC protocol number 17–178). All procedures were done in accordance with the relevant guidelines (ARRIVE Essential 10) and followed regulations for animal experimentation.

### Animals

Ninety*-*healthy Sprague Dawley rats (3–5 weeks old, 150–250 g, Charles River Laboratory) were included for the cardiomyocytes isolation, western blot analysis, skinned cardiac fiber, and ATPase study. To avoid gender and hormonal effect on cardiac function, only male rats were used. The calculated priori sample size was calculated using Gpower® software based on the previous experiments detecting the peak contraction of the cardiomyocytes perfusing with propranolol and isoproterenol^12^. We used 6 samples following the smallest number of samples used in that experiment^12^. No inclusion and exclusion criteria of animals were set in this study.

### Isolation, purification, and identification of β-CTX

Lyophilized Thai king cobra venom (TKCV) was purchased from Queen Saovabha Memorial Institute (QSMI), Bangkok, Thailand. β-CTX was isolated and purified from the TKCV by the sequential use of reverse phase following with cation exchange chromatography as previously described^3^. The presence of the compound was confirmed using SDS-PAGE and automated Edman’s degradation N-terminal sequencer (PPSQ™-33B, Shimadzu®).

### Cells isolation and simultaneous measurement of contraction and calcium profiles

The effect of the β-CTX was directly tested on cardiomyocytes to investigate the alteration in Ca^2+^ and cardiac mechanics. A total of 72 rats were used for cardiomyocyte isolation. The adult rat ventricular myocyte isolation and the measurement of the cellular functions were following the previous publication^3^. Briefly, the heart and aorta were cut and perfused with the perfusion solution (in mM: NaCl 133.5, KCl 4, NaH_2_PO_4_ 1.2, HEPES 100, MgSO_4_ 1.2, dextrose 33, and 0.1% bovine serum albumin), then switched to the enzyme solution (perfusion solution containing 0.025% collagenase II (Worthington®), 0.03% protease XIV (Sigma®) and 20 µM of CaCl_2_). After the manual and enzymatic digestion, reintroducition of calcium was then gradually performed until reached 1 μM of CaCl_2_. Three replicate of cardiomyocytes were used in each rat. After the cell isolation, cardiomyocytes were conducted in 2 different conditions, with the presence of isoproterenol (ISO) and forskolin (FSK). In the ISO stimulation experiment, animals were divided into 6 experimental groups receiving control solution (in mM: NaCl 133.5, KCl 4, NaH_2_PO_4_ 1.2, HEPES 100, MgSO_4_ 1.2, dextrose 33, CaCl_2_ 1.8), 0.3 µM propranolol (PP), 0.3 µM β-cardiotoxin (β-CTX), 1 µM ISO, 0.3 µM PP and 1 µM ISO (PP + ISO), and 0.3 µM β-CTX and 1 µM ISO (β-CTX + ISO). Each group contained 6 rats. In the FSK stimulation experiment, rats were also divided into 6 experimental groups with 6 rats per group (control solution, 0.3 µM PP, 0.3 µM β-CTX, 3 µM FSK, 0.3 PP with 3 µM FSK (PP + FSK), and 0.3 µM β-CTX with 3 µM FSK (β-CTX + FSK)). The perfusion rate of the treatment was set at 0.5 mL/min for 8 min until the effect becomes stable as previously described^3^.Simultaneously measurement of cardiac mechanical functions as well as Ca^2+^ homeostatic profiles, including myocyte length shortening, shortening velocity (+ dL/dt), relaxing index (τ), re-lengthening velocity (-dL/dt), peak Ca^2+^-transient (CaT) and Ca^2+^-decaying (τ_Ca_), were conducted. Data were calculated from 8–10 contractions in each cell. All parameters were normalized as percentage change from their baseline.

### ProQ® diamond staining

We screened the appearance of any changes in cardiac myofilament phosphorylation affected by β-CTX using the commercial protein phosphorylated stain (ProQ® diamond). Cardiomyocytes were isolated from six rats to perform the phosphorylation study. After the cardiomyocyte isolation process, cells were separated into 6 groups, treated with control, PP, β-CTX, ISO, PP + ISO, and β-CTX + ISO. Following the 8-min incubation of each treatment, cardiac myofibrils were isolated by modifying from previous report^48^. Briefly, cells were homogenized using Duall plastic homogenizing pestle in the standard relaxation buffer (SRB; in mM: KCl 75, Imidazole 10, MgCl_2_ 2, EGTA 2, and NaN_3_ 1) containing 1% Triton-X 100. Pellets were washed with normal SRB containing protease inhibitor (Sigma®), phosphatase inhibitor (Calbiochem®), and Calyculin A (Invitrogen™). Finally, pellets were solubilized in the industrial sample buffer (ISB; 8 M urea, 2 M thiourea, 0.05 M Tris, 75 mM DTT, 3% SDS, and 0.005% bromophenol blue; pH 6.8) before use. Loadings were divided into two conditions, basal state (control, β-CTX, PP, ISO) and the ISO-induction (control, ISO, β-CTX + ISO, PP + ISO). Myofibril preparations were electrophoresed in 15% SDS-PAGE, 200 V, 90 min. The gel was then stained with a phosphoprotein staining kit (ProQ® Diamond, Invitrogen™) and the total phosphorylation of protein bands was detected under ChemiDoc™ MP imager (BioRad®). Following the phosphorylated bands' measurement, gels were incubated with Coomassie brilliant blue stain (Biosafe™ Coomassie G-250, BioRad®) to acquire total protein expression. Band intensities were analyzed using ImageLab™ 6.0.1 software (BioRad®) and all phosphorylated bands were normalized to the total protein.

### Western blot analysis

Following the rat ventricular myocytes isolation from six rats, cells were equally divided into 6 groups as the same as the previous protocol (control, PP, β-CTX, ISO, PP + ISO, and β-CTX + ISO). After the incubation for 8 min, they were centrifuged at 200xg, 4 °C for 3 min, and the supernatant was discarded. Cells in each group were added with ISB and homogenized. Lysates were loaded into 15% SDS-PAGE and electrophoresed at 200 V, 90 min. Following the electrophoresis, gels were then transferred onto 0.22 µm PVDF membranes (Immobilon®). Non-specific proteins were blocked with 5% non-fat dry milk in Tris-Base Saline-Tween Solution (TBS-T; in mM: 50 Tris-Base, 200 NaCl, 0.1% (v/v) Tween-20, pH 7.5). To study the alteration in β-adrenergic protein phosphorylation, we incubated membranes overnight at 4 °C with primary antibodies for p-cMyBP-C (Ser279, Ser288, and Ser308; courtesy to Sakthivel Saddayapan), total cMyBP-C (courtesy to Rick Moss), p-cTnI (Ser23/24; Cell Signaling Technology® 4004B), total cTnI (Fitzgerald® 10R-T123k), p-PLN (Ser16; Millipore™ 07,052, and Thr17; Badrilla™ A010-13AP), total PLN (Badrilla™ A010-14) and total SERCa2a (Badrilla™ A010-23). After the overnight incubation, secondary antibodies (goat anti-rabbit; Cell Signaling Technology® 7074, or horse anti-mouse; Cell Signaling Technology® 7076, conjugated with horseradish peroxidase), were added. The blots were developed by enhanced chemiluminescence (Clarity™, BioRad®) using the ChemiDoc™ MP imager (BioRad®). Band densities were analyzed using ImageLab™ 6.0.1 software (BioRad®) and all phosphorylated bands were normalized to the total protein and β-actin.

### Preparation of detergent-extracted (skinned) fiber bundles and the force-pCa relationship determination

A total of three rats were used for the detergent-extracted fiber experiment. The isolation of the rat papillary muscle and the measurement of force generated responsive to pCa were described previously^49^. Briefly, left papillary muscles were dissected from the heart. Muscle fibers were gently skinned and separated under a detergent (1% Triton-X) containing solution. Eleven skinned fibers were further used to find the force-pCa relationship. The fibers were finally mounted on the force transducer. Eight selected pCa solutions (4.50, 5.50, 5.75, 5.85, 6.00, 6.25, 6.50, and 7.00) were used in this experiment. Fibers were incubated in each pCa solution for 8 min before measurement or until the effect becomes stable and plateau. Force generation was recorded and compared between fibers in normal high relaxation (HR) buffer (in mM: EGTA 10, KCl 41.89, MgCl_2_ 6.57, BES 100, ATP 6.22, creatine phosphate 10, and sodium azide 5), as control, and the same fiber receiving HR containing 1 µM of β-CTX. Maximal velocity, pCa_50_, and Hill’s coeffeicient were extrapolated from the records.

### Myofibrils preparation and evaluation of S1 actomyosin ATPase activity

Twelve rats were divided into two experimental groups (i.e. control and β-CTX). Methods for isolating rat ventricular myofibrils were modified from previous publications^50^. Briefly, rat ventricular tissue was cut and homogenized in SRB containing 1 mg/mL of Collagenase II (Worthington®) following by SRB with 1% v/v Triton X-100. Myofibrils were transferred into A-70 buffer (in mM: NaCl 70, MgCl_2_ 10, and MOPS 40). Ca^2+^-activation of myofibrillar activity was measured using a malachite green assay as previously described^51^. In brief, myofibril suspensions were incubated in various Ca^2+^ concentrations as expressed in pCa (−log [Ca^2+^]) with either control (A-70 buffer; n = 6) or 1 μM of β-CTX (n = 5), for 8 min before the reaction began. Of note, one ATPase measurement of the β-CTX was excluded due to the unusually ATPase activity of the myofibrils. Twelve different points of pCa were selected in the study (4.03, 4.58, 4.87, 5.09, 5.25, 5.48, 5.82, 6.06, 6.25, 6.60, 7.38 and 7.70). The reaction began with the addition of 1.67 mM of ATP. The production of inorganic phosphate (P_i_) was evaluated by stopping the reaction in perchlorate and incubated with a malachite green solution at 27 °C for 30 min and detected at the absorbance of 655 nm. The ATPase activity was calculated by the amount of P_i_ produced at each time point.

### Statistical analysis

To test if the data in each experiment were normally distributed, the Kolmogorov–Smirnov test with Dallal-Wilkinson-Lillie were used. All data fell into Gaussian distribution. One-way ANOVA, followed by Tukey’s post hoc test, was conducted to compare cardiomyocyte function parameters in cardiomyocytes amongst treatments as indicated in Figs. 1 and 2. The phosphorylation of proteins was assessed using one-way ANOVA followed by Tukey’s test as multiple comparisons as shown in Figs. 3, 4 and 5. Sigmoidal fit-curve of concentration responses was plotted among force-pCa study and ATPase activity. Variables obtained from the former were compared between before and after receiving β-CTX by using paired t-test (Fig. 6); whereas the latter was assessed using an unpaired student t-test (Fig. 7). All data were represented in mean ± S.E.M. and significance was considered at *p* < 0.05. All statistical analysis was performed under commercial software (GraphPad® Prism).

## Supplementary Information

Supplementary Information 1.
