# Supplementary material for: Negative inotropic mechanisms of β-cardiotoxin in cardiomyocytes by depression of myofilament ATPase activity without activation of the classical β-adrenergic pathway
Source: Sci Rep. 2021 Oct 27;11:21154. doi: 10.1038/s41598-021-00282-x (PMC8551325; doi:10.1038/s41598-021-00282-x)
Supplement: Supplementary file 1 — Supplementary Information 1. [file 41598_2021_282_MOESM1_ESM.docx]

**Supplementary information**

**Negative Inotropic Mechanisms of β-cardiotoxin in Cardiomyocytes by Depression of Myofilament ATPase Activity without Activation of** **the** **classical β-adrenergic pathway**

Tuchakorn Lertwanakarn^1^, Montamas Suntravat^2,3^, Elda E Sánchez^2,3^, Beata M Wolska^4,5^, R John Solaro^4^, Pieter P de Tombe^4,6^, Kittipong Tachampa^1,*

1^ Department of Physiology, Faculty of Veterinary Science, Chulalongkorn University, Thailand, ^2^ National Natural Toxins Research Center, Texas-A&M University-Kingsville, TX, USA., ^3^ Department of Chemistry, Texas A&M University-Kingsville, TX, USA., ^4^ Department of Physiology and Biophysics, the University of Illinois at Chicago, IL, USA., ^5^ Department of Medicine, the University of Illinois at Chicago, IL, USA., ^6^ Phymedexp, Université de Montpellier, Inserm, CNRS, Montpellier, France.

** Corresponding author; email: Kittipong.T@chula.ac.th/*


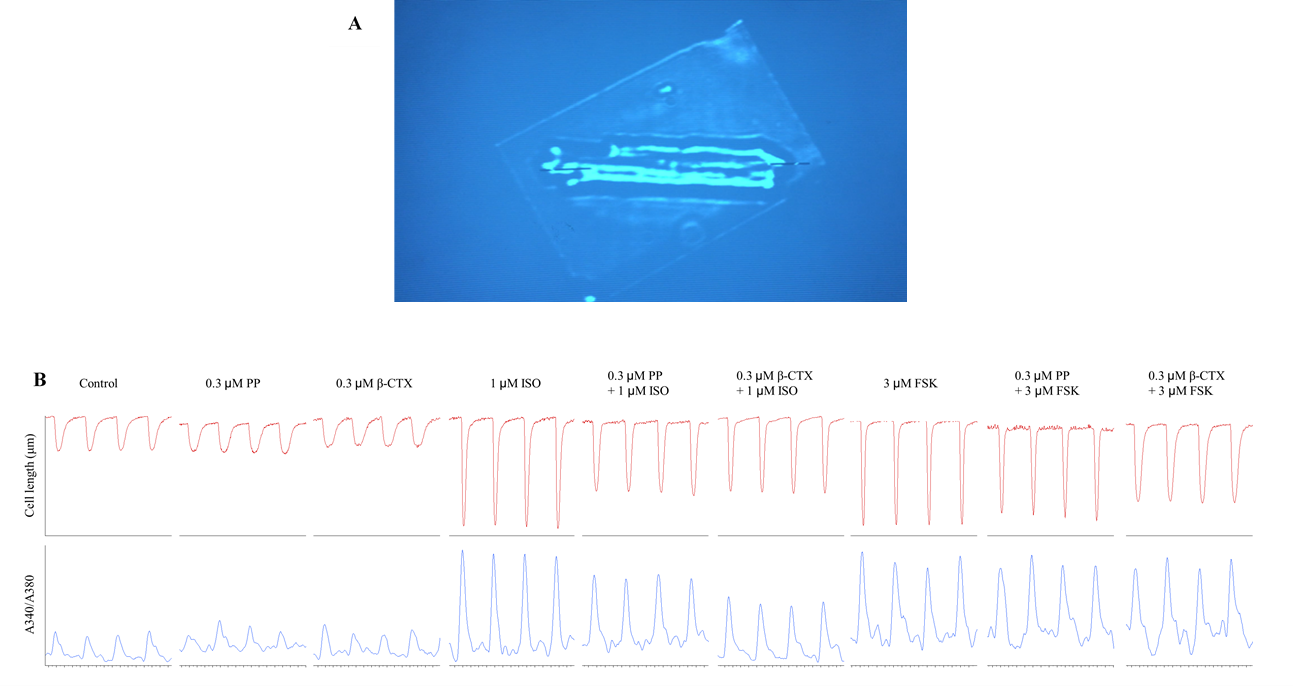


**Figure S1. Supplementary for figure 1 and 2:** (A) Illustration of isolated cardiomyocyte measurement using edge detection system. (B) Representative of raw tracings of cardiomyocytes showing cell contraction (upper panel), and calcium transient profile (lower panel)


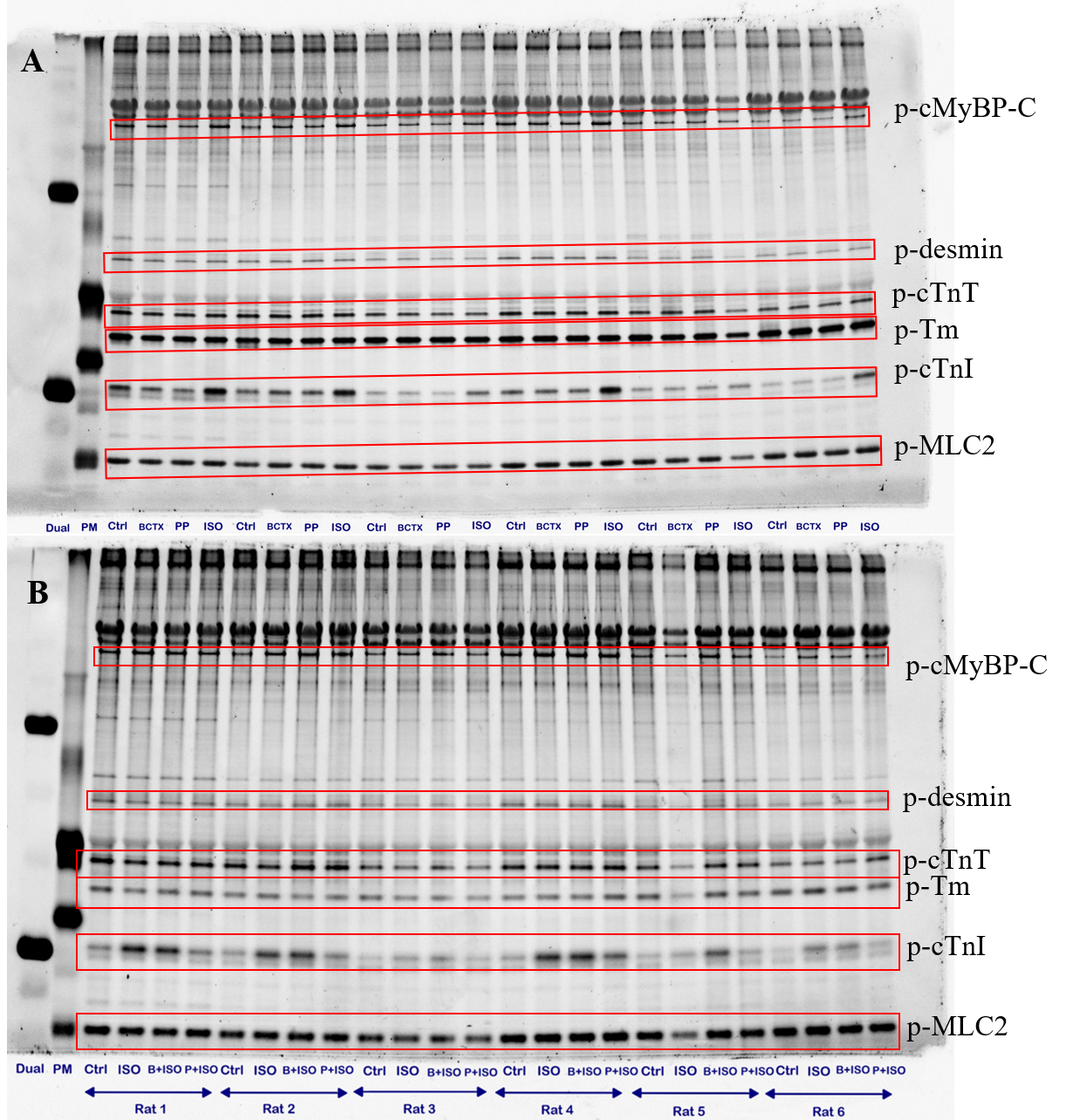


**Figure S2: Supplementary figure for figure 3.** 15% Polyacrylamide gel of cardiac myofilament proteins stained with ProQ ^®^. The total levels of phosphorylation of cardiac myosin binding protein-C (cMyBP-C), desmin, cardiac troponin T (cTnI), tropomyosin (Tm), cardiac troponin I (cTNI), and myosin light chain-2 (MLC2) (n=6 each). Each protein is compared at the basal state (A), and ISO-induced condition (B). Dual; Precision plus protein dual color standards (BioRad^®^), PM; Pepperminstick^TM^ Phosphoprotein molecular weight standards, Ctrl; control group, BCTX; β-CTX, PP; propranolol, ISO; isoproterenol, B+ISO; β-CTX with isoproterenol, P+ISO, propranolol with isoproterenol.


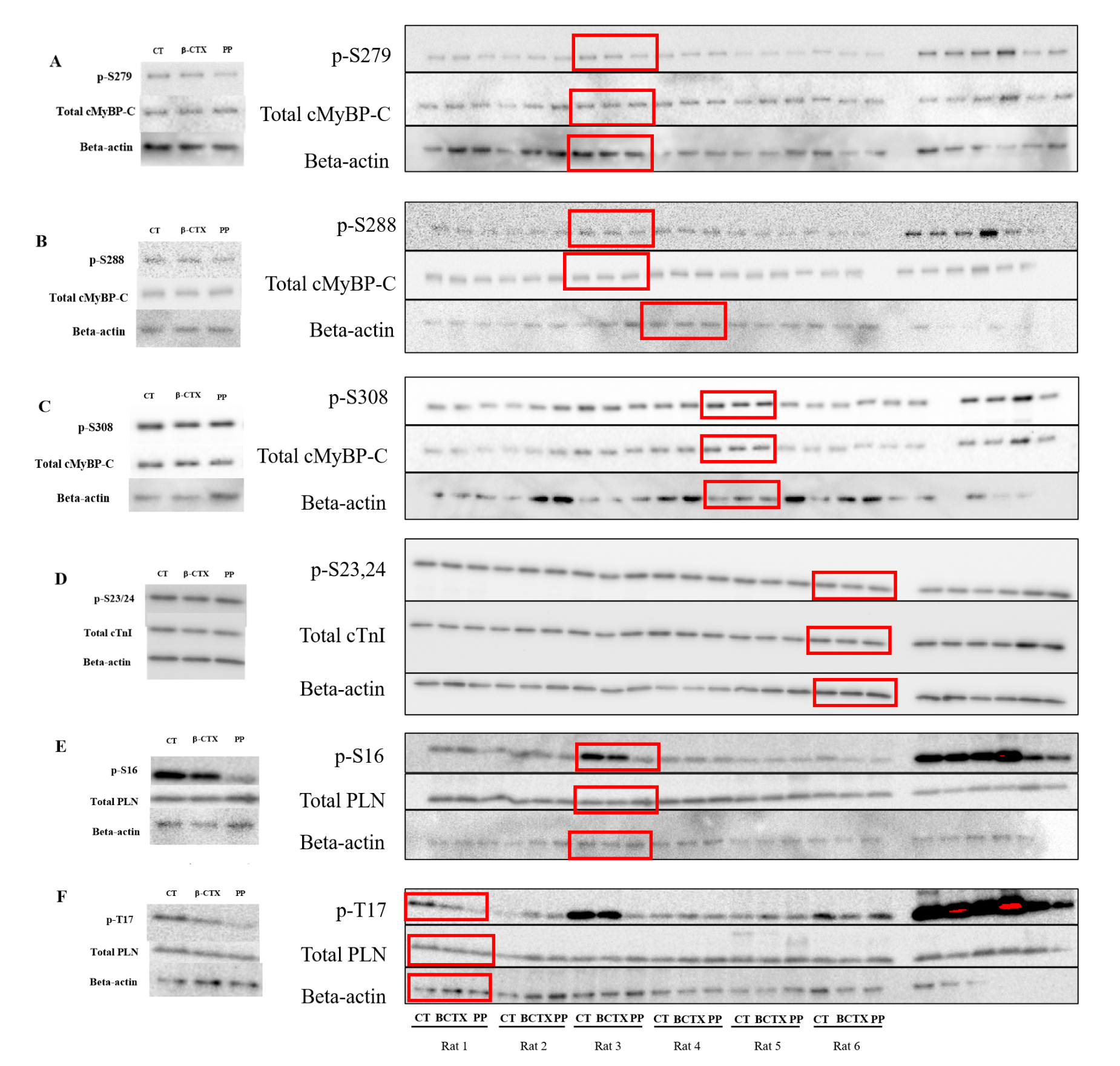


**Figure S3: Supplementary for figure 4:** Western blots shown in the main text (left panel), and blots with cropping line (right panel) of (A) S279, (B) S288, (C) S308 of cMyBP-C, (D) S23,24 of cTnI, (E) S16 and (F) T17 of PLN at the basal state. CT: control, BCTX:β-CTX , PP: propranolol (n=6 each)


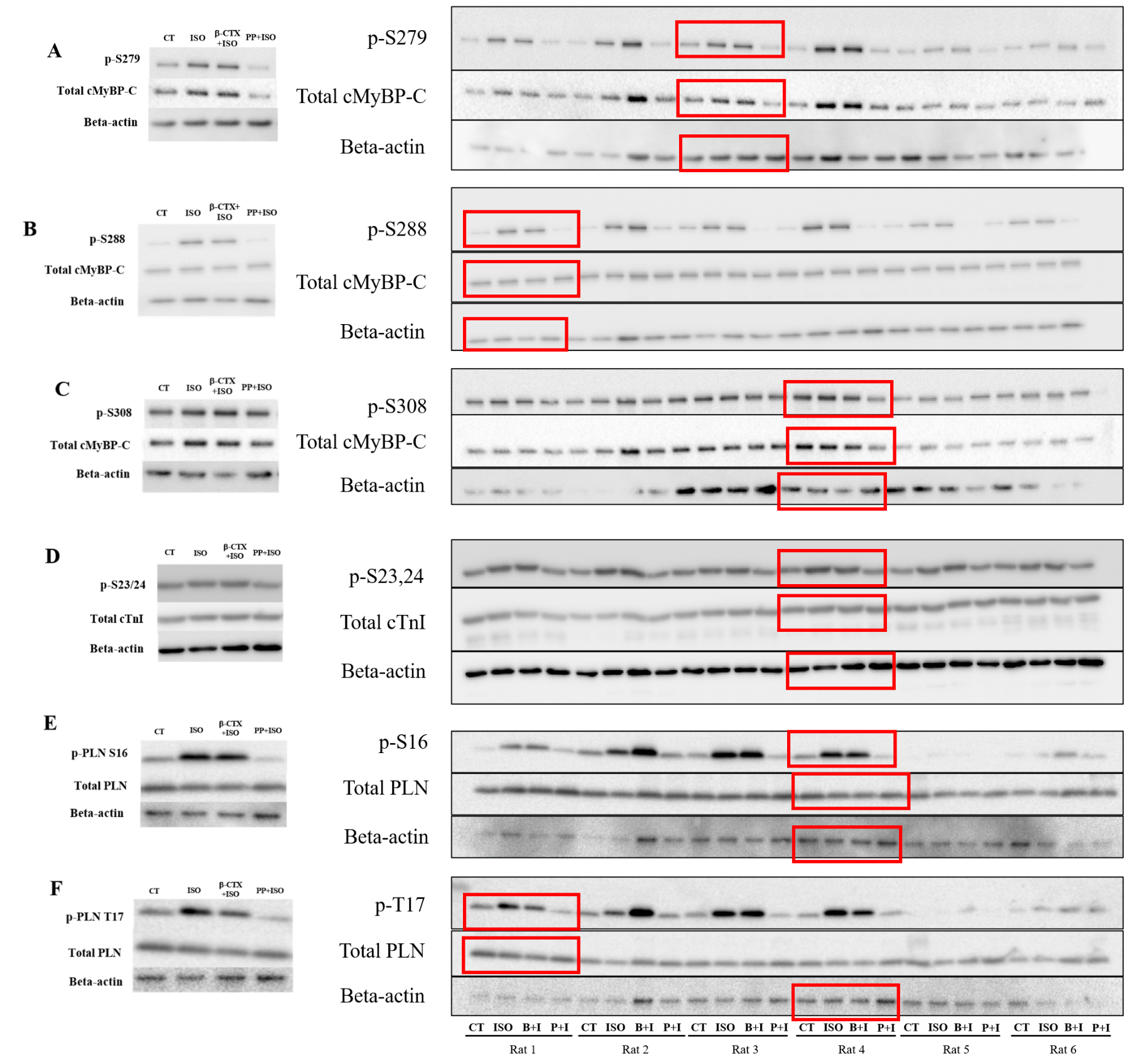


**Figure S4: Supplementary for figure 5:** Western blots shown in the main text (left panel), and blots with cropping line (right panel) of (A) S279, (B) S288, (C) S308 of cMyBP-C, (D) S23,24 of cTnI, (E) S16 and (F) T17 of PLN during the ISO-stimulating condition. CT: control, ISO: isoproterenol, B+I: β-CTX+ISO, P+I: propranolol+ISO (n=6 each).


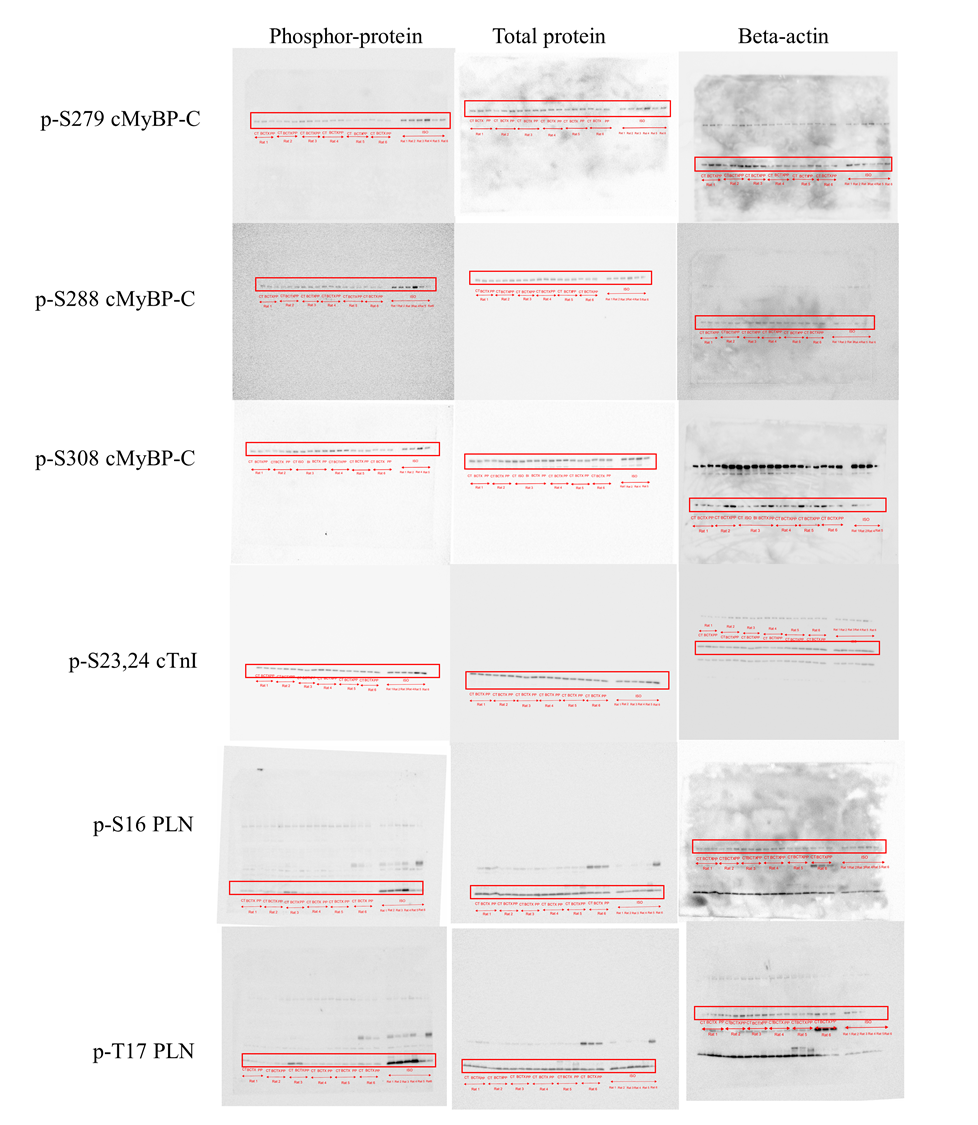


**Figure S5: Supplementary full-length blot shown in figure S3.** The cropped area represented the images shown in figure S3. There are three conditions at this basal state, control (CT), β-CTX-, and propranolol (PP)-treated cells (n=6 each). Notably, each membrane was exposed three times, using phosphor-protein, the total of that protein, and the hose keeping beta-actin antibodies, respectively.


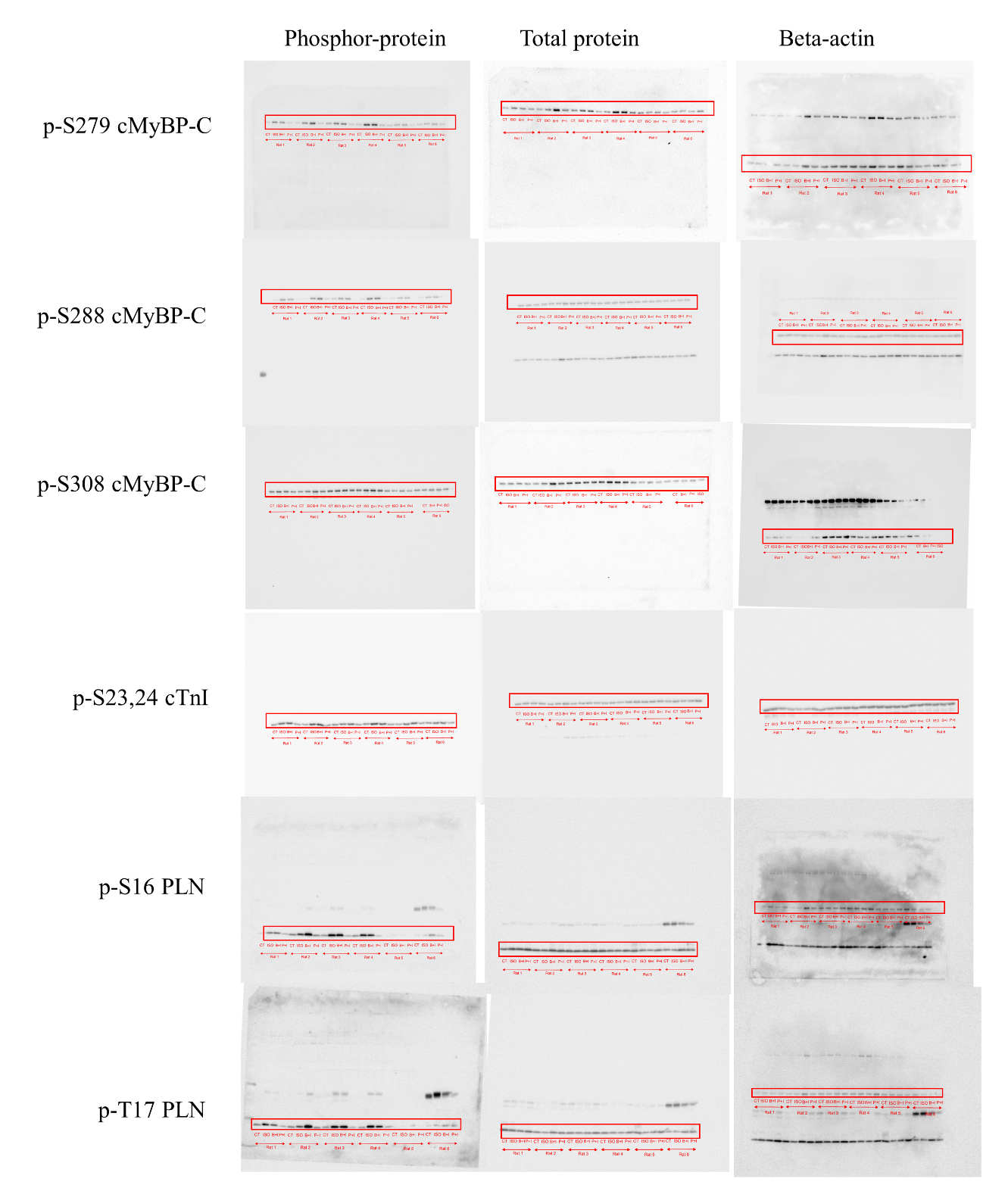
**Figure S6. The supplementary full-length blot is shown in figure S4.** The cropped area represented the images shown in figure S4. There are four conditions including, control (CT), isoproterenol (ISO), β-CTX with ISO (B+I), and propranolol with ISO (P+I)-treated cells (n=6 each). Notably, each membrane was exposed three times, using a phosphor-protein antibody, the total of that protein, and the hose keeping beta-actin, respectively.

**Table S 1.** Supplemental data for figure 1. Percentage changes from the baseline of each parameter (n=6 each) are presented in mean ± S.E.M.

| **Parameters** | **CTRL** | **PP** | **BCTX** | **ISO** | **PP+ISO** | **BCTX+ISO** |
| --- | --- | --- | --- | --- | --- | --- |
| **Shortening** | 100.00^a^ | 72.35±3.75^a^ | 56.07±3.16^a^ | 411.20±16.36^b^ | 311.21±20.03^c^ | 311.75±11.04^c^ |
| **+dL/dt** | 100.00^a^ | 7.03±3.64^a^ | 55.68±1.54^a^ | 817.94±110.77^b^ | 496.60±27.66^c^ | 410.32±38.51^c^ |
| **-dL/dt** | 100.00^a^ | 71.55±2.65^a^ | 49.91±37.48^a^ | 911.45±120.29^b^ | 499.35±37.48^c^ | 471.382±21.26^c^ |
| **Tau** | 100.00^a^ | 110.55±2.70^a^ | 141.31±12.07^b^ | 51.02±1.66^c^ | 41.70±2.69^c^ | 62.96±1.81^c^ |
| **CaT** | 100.00^a^ | 95.12±2.30^a^ | 112.852±3.24^a^ | 465.38±37.58^b^ | 319.92±17.80^c^ | 257.79±8.45^c^ |
| **Ca decay** | 100.00^a^ | 118.23±8.58^a^ | 117.673±5.47^a^ | 39.71±1.49^b^ | 49.65±2.31^b^ | 66.88±4.97^b^ |

**Table S 2.** Supplemental data for figure 2. Percentage changes from the baseline of each parameter (n=6 each) are presented as mean S.E.M.

| **Parameters** | **CTRL** | **PP** | **BCTX** | **ISO** | **PP+ISO** | **BCTX+ISO** |
| --- | --- | --- | --- | --- | --- | --- |
| **Shortening** | 100.00^a^ | 76.83±5.59^a^ | 70.99±8.77^a^ | 353.90±76.96^b^ | 307.44±68^b^ | 227.48±24.72^a,b^ |
| **+dL/dt** | 100.00^a^ | 79.69±61.22^a^ | 76.48±4.35^a^ | 379.13±96.45^b^ | 397.37±93.92^b^ | 262.43±34.57^a,b^ |
| **-dL/dt** | 100.00^a^ | 70.18±8.02^a^ | 77.75±4.91^a^ | 542.34±145.49^b^ | 481.39±121.97^b^ | 314.51±54.14^a,b^ |
| **Tau** | 100.00^a^ | 107.48±4.76 | 108.47±6.52 | 90.13±7.70 | 80.66±13.92 | 82.03±9.03 |
| **CaT** | 100.00^a^ | 98.74±4.99^a^ | 92.64±10.60^a^ | 63.63±9.17^b^ | 60.84±6.15^b^ | 64.57±5.14^b^ |
| **Ca decay** | 100.00^a^ | 114.57±7.00^a^ | 104.41±6.77^a,b^ | 250.71±49.67^b,c^ | 235.61±20.67^c^ | 232.77±15.85^b,c^ |

**Table S 3.** Supplemental data for figure 3 and figure S2. Analysis of phosphorylation of myofibrillar proteins (n=6 each) is shown as mean ± S.E.M.

| **Phosphor-protein (ProQ)** | **CTRL** | **PP** | **BCTX** | **ISO** |
| --- | --- | --- | --- | --- |
| **p-cMyBP-C** | 0.087±0.011^a^ | 0.077±0.011^a^ | 0.064±0.013^a^ | 0.142±0.014^b^ |
| **p-Desmin** | 0.136±0.010 | 0.127±0.012 | 0.149±0.013 | 0.138±0.008 |
| **p-cTnT** | 0.098±0.002 | 0.099±0.005 | 0.100±0.004 | 0.100±0.002 |
| **p-Tm** | 0.149±0.008 | 0.142±0.006 | 0.146±0.004 | 0.147±0.008 |
| **p-cTnI** | 0.071±.014^a^ | 0.063±0.011^a^ | 0.061±0.011^a^ | 0.146±0.024^b^ |
| **p-MLC** | 0.087±0.008 | 0.083±0.009 | 0.086±0.010 | 0.081±.009 |

| **Phosphor-protein (ProQ)** | **CTRL** | **ISO** | **BCTX+ISO** | **PP+ISO** |
| --- | --- | --- | --- | --- |
| **p-cMyBP-C** | 0.155±0.010^a^ | 0.261±0.014^b^ | 0.240±0.021^b^ | 0.196±0.025^a^ |
| **p-Desmin** | 0.243±0.037 | 0.244±0.034 | 0.265±0.028 | 0.239±0.035 |
| **p-cTnT** | 0.180±0.005 | 0.176±0.009 | 0.189±0.005 | 0.189±0.013 |
| **p-Tm** | 0.144±0.008 | 0.140±0.008 | 0.142±0.009 | 0.137±0.007 |
| **p-cTnI** | 0.116±0.022^a^ | 0.345±0.053^b^ | 0.357±0.060^b^ | 0.167±0.022^a^ |
| **p-MLC** | 0.096±0.009 | 0.093±0.011 | 0.093±0.009 | 0.089±0.010 |

**Table S 4.** Supplemental data for figure 4. The ratio between the phosphor- and total protein were compared among treatments (n=6 each). Data are shown in mean ± S.E.M.

| **Phosphor/Total protein** | **CTRL** | **BCTX** | **PP** |
| --- | --- | --- | --- |
| **p-S279/cMyBP-C** | 0.666±0.119 | 0.631±0.154 | 0.375±0.070 |
| **p-S288/cMyBP-C** | 1.541±0.103 | 1.344±0.104 | 1.225±0.162 |
| **p-S288/cMyBP-C** | 1.048±0.080 | 1.081±0.118 | 1.095±0.093 |
| **p-S23-24/cTnI** | 1.602±0.144 | 1.600±0.150 | 1.605±0.132 |
| **p-S16/PLN** | 0.282±0.048 | 0.257±0.050 | 0.218±0.034 |
| **p-T17/PLN** | 0.753±0.090 | 0.625±0.042 | 0.484±0.084 |

**Table S 5.** Supplemental data for figure 5. The ratio between the phorphor- and total protein were compared among groups (n=6 each). Data are presented as mean S.E.M.

| **Phosphor/Total protein** | **CTRL** | **ISO** | **BCTX+ISO** | **PP+ISO** |
| --- | --- | --- | --- | --- |
| **p-S279/cMyBP-C** | 0.461±0.068^a^ | 1.171±0.082^b^ | 1.189±0.104^b^ | 0.365±.048^a^ |
| **p-S288/cMyBP-C** | 0.269±0.060^a^ | 1.515±0.176^b^ | 1.557±0.203^b^ | 0.140±0.039^a^ |
| **p-S288/cMyBP-C** | 4.233±0.227 | 4.839±0.489 | 4.713±0.411 | 3.921±.181 |
| **p-S23-24/cTnI** | 0.642±0.081 | 0.778±.038 | 0.959±0.145 | 0.601±0.101 |
| **p-S16/PLN** | 0.305±0.093^a^ | 1.281±0.436^b^ | 1.447±0.402^b^ | 0.164±0.005^a^ |
| **p-T17/PLN** | 0.585±0.105^a^ | 2.702±0.827^b^ | 2.283±0.834^b^ | 0.512±0.103^a^ |

**Table S 6.** Supplemental data for figure 7. PArameters from ATPase activity measurement was compared between control (n=6) and b-CTX group (n=5). Data are represented in mean ± S.E.M.

| **Paramaeters** | **CTRL** | **BCTX** | ***p*-value** |
| --- | --- | --- | --- |
| **Maximal ATPase activity** | 0.980±0.012 | 0.922±0.003 | 0.004 |
| **pCa50** | 5.685±0.027 | 5.574±0.012 | 0.0128 |
| **Hill’s coefficient** | 0.961±0.086 | 1.042±0.088 | 0.5453 |
